# An atypical presentation of autopsy confirmed rapidly progressive early onset Alzheimer's disease with borderline CSF biomarkers: A case study

**DOI:** 10.1002/alz70856_107697

**Published:** 2026-01-09

**Authors:** Kailey Zajicek, Allison Lapins, Molly A Mather, Tamar Gefen, Rudolph J Castellani, Sandra Weintraub, Robert J. Vassar, Marsel Mesulam

**Affiliations:** ^1^ Mesulam Center for Cognitive Neurology and Alzheimer's Disease, Chicago, IL, USA; ^2^ Northwestern University Feinberg School of Medicine, Chicago, IL, USA; ^3^ Mesulam Center for Cognitive Neurology & Alzheimer's Disease, Chicago, IL, USA

## Abstract

**Background:**

Most patients clinically diagnosed with dementia due to Alzheimer's disease (AD) are over the age of 65 and exhibit a slowly progressive amnestic clinical syndrome. In contrast, those diagnosed clinically with Frontotemporal Dementia (FTD) tend to have younger age of onset and clinical presentations with behavioral and/or language decline. With the advent of cerebrospinal fluid (CSF) AD biomarkers with high sensitivity and specificity, differentiation between dementia due to AD vs FTD can be made with significantly more confidence during life. Though CSF biomarker testing for AD has excellent diagnostic accuracy for straightforwardly positive or negative results, guidance for interpretation of “borderline” or “indeterminate” results, which is common in clinical practice, is limited.

**Method:**

We summarize available clinical, biomarker, and autopsy data for a 56‐year‐old woman enrolled into the Northwestern University Alzheimer's Disease Research Center with a 3‐year history of rapidly progressive decline in language and behavior, borderline cerebrospinal fluid (CSF) biomarkers for AD, but was found to have high level Alzheimer's Disease Neuropathic Change (ADNC) at autopsy.

**Results:**

CSF testing for AD occurred when the patient was in a severe stage of dementia, 1.5 years into the disease process and 15 months before death. Whereas the Aβ_42_/total tau index (ATI) value fell within the AD range, phosphorylated tau (*p*‐tau) was only borderline elevated (Ab_42_ = 380 pg/mL, total tau = 414 pg/mL, ATI = 0.62, *p*‐tau = 58.8 pg/mL). Genetic testing was negative for known mutations that cause FTD. The neuropathological findings at autopsy indicated high ADNC. There were incidental Lewy bodies in the brainstem, but no significant FTLD‐tau or TDP‐43 neuropathologic changes.

**Conclusion:**

This case illustrates an atypical, rapidly progressive clinical presentation of autopsy‐confirmed AD with CSF biomarkers for AD in the “borderline” range relatively close to death. In clinical practice, cases where biomarkers are “borderline” are not uncommon and pose challenges to differentiating between dementias due to different underlying neurodegenerative diseases. Learning from atypical presentations such as this one may augment diagnostic accuracy and clinical disease management. Further research is needed to establish correspondence of “borderline” or “indeterminate” CSF biomarkers to neuropathological findings seen at autopsy.

